# User Preferences and Design Recommendations for an mHealth App to Promote Cystic Fibrosis Self-Management

**DOI:** 10.2196/mhealth.3599

**Published:** 2014-10-24

**Authors:** Marisa E Hilliard, Amy Hahn, Alana K Ridge, Michelle N Eakin, Kristin A Riekert

**Affiliations:** ^1^Baylor College of MedicineDepartment of PediatricsHouston, TXUnited States; ^2^Nationwide Children's HospitalColumbus, OHUnited States; ^3^Johns Hopkins University School of MedicineDepartment of MedicineBaltimore, MDUnited States

**Keywords:** cystic fibrosis, qualitative research, mobile health

## Abstract

**Background:**

mHealth apps hold potential to provide automated, tailored support for treatment adherence among individuals with chronic medical conditions. Yet relatively little empirical research has guided app development and end users are infrequently involved in designing the app features or functions that would best suit their needs. Self-management apps may be particularly useful for people with chronic conditions like cystic fibrosis (CF) that have complex, demanding regimens.

**Objective:**

The aim of this mixed-methods study was to involve individuals with CF in guiding the development of engaging, effective, user-friendly adherence promotion apps that meet their preferences and self-management needs.

**Methods:**

Adults with CF (n=16, aged 21-48 years, 50% male) provided quantitative data via a secure Web survey and qualitative data via semi-structured telephone interviews regarding previous experiences using apps in general and for health, and preferred and unwanted features of potential future apps to support CF self-management.

**Results:**

Participants were smartphone users who reported sending or receiving text messages (93%, 14/15) or emails (80%, 12/15) on their smartphone or device every day, and 87% (13/15) said it would be somewhat or very hard to give up their smartphone. Approximately one-half (53%, 8/15) reported having health apps, all diet/weight-related, yet many reported that existing nutrition apps were not well-suited for CF management. Participants wanted apps to support CF self-management with characteristics such as having multiple rather than single functions (eg, simple alarms), being specific to CF, and minimizing user burden. Common themes for desired CF app features were having information at one’s fingertips, automation of disease management activities such as pharmacy refills, integration with smartphones’ technological capabilities, enhancing communication with health care team, and facilitating socialization within the CF community. Opinions were mixed regarding gamification and earning rewards or prizes. Participants emphasized the need for customization options to meet individual preferences and disease management goals.

**Conclusions:**

Unique capabilities of emerging smartphone technologies (eg, social networking integration, movement and location detection, integrated sensors, or electronic monitors) make many of these requests possible. Involving end users in all stages of mHealth app development and collaborating with technology experts and the health care system may result in apps that maintain engagement, improve integration and automation, and ultimately impact self-management and health outcomes.

## Introduction

### Background

Despite the well-documented need for improved adherence to prescribed medical treatments for chronic health conditions [1,2], interventions targeting treatment adherence have had modest results, and typically demonstrate small effect sizes [3]. Behavioral interventions tend to be time- and resource-intensive and are disconnected from standard medical care. Researchers have attempted to address these challenges by using technology for adherence promotion, initially through educational or interactive videogames [4] and more recently via text messaging-based medication reminder systems [5,6]. Unfortunately, barriers including the length of time it takes to develop and evaluate technology-based interventions using a static platform can result in programs that are outdated, obsolete, and clinically ineffective [7]. Behavioral interventions that use technologies or are delivered via the Internet have begun to address some of these common limitations, with evidence of improved treatment adherence [8]. Apps for smartphones and other mobile devices represent a promising avenue for further innovation in mHealth adherence promotion.

The use of mobile technologies is widespread and growing rapidly, and is increasingly intersecting with individuals’ health management. Smartphone ownership continues to rise: 91% of American adults now have a cell phone and over half (56%) own a smartphone [9]. A 2012 Pew Internet poll reported that one-half of smartphone owners look up health information on their phones and 19% have at least one health-related app [10]. Consistent with this growth, there has been a surge in mobile technologies to support health, and it is not surprising that researchers are increasing their efforts to integrate mHealth technology into adherence promotion interventions.

### Trends in mHealth Intervention Research

Early evidence suggests that there is a high level of patient interest in mHealth interventions [11-13]. Text messaging has been an early focus of mHealth research and has shown modest, short-term benefits for adherence in multiple illness groups [14-19]. However, in contrast to the relatively common use of text messaging in adherence promotion research, dissemination has been slow: fewer than 10% of cell phone owners have signed up to receive health-related text messages [10], of which only a portion are related to adherence promotion. Moreover, as technologies advance, text messaging reminders may not be dynamic or interactive enough to sufficiently engage participants and impact health behaviors. There is a need to develop apps that utilize the advanced technological capabilities of smartphones to more effectively integrate behavioral strategies known to improve treatment adherence for people with chronic medical conditions [20].

Advances in mobile technologies permit researchers to develop automated mHealth interventions targeted to individuals’ specific treatment regimens and personal characteristics that can facilitate self-management in multiple ways [21]. For example, smartphones with embedded or external sensors can track and provide real-time feedback on the occurrence of particular behaviors or events, context awareness can trigger delivery of behavioral intervention at appropriate times or locations, and social networking functions can facilitate communication between patients and providers or among family members [21-23]. Integrating interactive features at this level has the potential to boost the efficacy of mHealth interventions in diseases with complex daily management needs. However, currently available mHealth apps largely focus on educational content or provide basic monitoring or reminding functions [24] and tend not to use the unique features and capabilities of mobile platforms (eg, context sensors, graphical data visualization, real-time data collection, and feedback) to their maximum potential [23,24]. With so many possibilities, a critical first step is to determine which design features have sufficient empirical evidence of efficacy, user interest, and potential to support chronic disease self-management. To maximize engagement with the software, it is essential for app developers to involve participants in the process of developing content and features that appeal to them [25]. In recent years, this idea of “user-centered design” [26] has begun to gain strength, and a handful of studies have reported user preferences for apps to promote health behaviors [27-30].

### Study Aims

Thus, the goal of this study was to involve individuals with cystic fibrosis (CF) in guiding the development of future mHealth apps for adherence promotion via mixed-methods research. Given the ever-expanding capabilities of smartphone technology, our purpose was to identify which potential app features would be of greatest interest to individuals with CF to support their daily disease management needs. We hypothesized that a primarily young adult sample of smartphone-owning individuals with CF would be interested in an app for smartphones or mobile devices to support disease management, and that they would recommend various strategies to maximize the relevance and utility of mHealth apps to support adherence to CF treatments.

## Methods

### Participants

Potentially eligible patients were identified from the patient roster of the adult CF clinic at a large, urban hospital in the mid-Atlantic United States. Clinic staff emailed all patients with email addresses on file to introduce the study and to offer an opportunity to opt-out of being recruited to participate. Study staff then telephoned potentially eligible participants to verify eligibility, describe the study in detail, answer questions, and obtain verbal informed consent. Eligibility requirements included age 18 years or above, diagnosis of CF, currently treated at the hospital’s adult clinic, prescribed at least one pulmonary medication (eg, inhaled mucolytic, inhaled or oral antibiotic therapy, hypertonic saline), and own or use a mobile device (eg, smartphone, tablet). For informational purposes, 235 clinic patients were emailed general information about the study, with an option to call the study team to opt out of being recruited for the study. Two people opted out of further contact at this stage. To actively recruit potential participants, study staff attempted to contact 72 patients randomly selected from the list of people who had been emailed. Of those we attempted to contact, 44 were not able to be reached by telephone. Of the 28 with whom we made contact, four were ineligible (one not diagnosed with CF, two not prescribed any CF medications, and one not a smartphone owner) and two declined to participate due to time restrictions and feeling that “my CF is atypical”. The remaining 22 indicated interest in participating and 19 consented to participate (three were unable to be reached to obtain consent after indicating interest); 16 people (84%) subsequently provided qualitative data via telephone interview (technical problems resulted in 15 audiotaped interviews available for coding), and 15 (79%) completed an online survey. Recruitment was stopped once sufficient data had been collected for the study team to identify the core concepts and themes, without additional information being gained from new participants; this is known as reaching thematic saturation [31].

### Cystic Fibrosis as an Exemplar Condition for mHealth Apps

Apps for health behavior promotion may be most appealing and useful for people who need help or support managing a chronic medical condition [28]. Yet, little is known about the needs and wants of people with most chronic conditions [27,29].

CF is an exemplar chronic condition for which mHealth apps could have potential relevance. CF is a chronic, progressive, obstructive pulmonary disease with a complicated and demanding treatment regimen. For people with CF, decreased lung function, pulmonary exacerbations, and being underweight are associated with increased mortality and morbidity, and with decreased quality of life. Just 35 years ago, a child born with CF had a median predicted survival of 14 years; today it is over 40 years and more than half of those with CF are now adults [32]. The increased survival of individuals with CF is attributable to advances in the medical treatment of CF; however, the complex CF management regimen is extremely demanding. On average, it takes adults with CF nearly 2 hours to complete seven therapies per day [33]. A typical daily treatment regimen includes chest physiotherapy and inhaled medications to break down and expel mucus from the lungs, and oral antibiotics and nebulized antibiotics (every other month) to prevent and treat infections. The majority of individuals with CF are also prescribed multiple daily vitamins, nutritional supplements, pancreatic enzymes to take with snacks and meals, and a diet high in calories, fat, and sodium. As people with CF age, they are a greater risk for diagnosis of CF-related diabetes that requires carbohydrate tracking. Most prescribed CF medications are classified as specialty drugs, and obtaining refills requires extra authorizations and paperwork that must be coordinated between clinicians, pharmacists, and insurers. Because of the risk of cross-infection, it is recommended that people with CF not have in-person contact with others with CF. Between these infection control guidelines and the relatively small population with CF, many people have never met another person with CF. This often leads to a sense of isolation and lack of social support from others who understand firsthand their treatment burden [34-36]. Because care is provided at specialty CF clinics, many individuals with CF travel long distances for routine care, making social support from providers and in-person adherence counseling challenging.

The motivation and organizational skills required to consistently follow this demanding regimen can lead to suboptimal CF management [37], which has implications for increased morbidity and health care utilization [38]. Given the widespread and growing use of smartphones and mobile devices, mHealth technologies represent a promising avenue for automated yet individually tailored interventions to support adherence to the complicated, time-consuming CF management regimen [14]. Moreover, the complex treatment needs of CF make it an exemplar condition for which to develop mHealth apps that could serve as a model for other chronic conditions.

### Procedures

Human subjects research approval was obtained from the institutional review board. After providing oral consent, participants completed a 30-45 minute semi-structured telephone interview with study staff. The interviews were digitally recorded and transcribed to facilitate coding and interpretation. Participants were also emailed a secure link to a password-protected Web-based survey to be completed within 2 weeks. Participants were compensated US $50 for completing the interview and online survey.

### Measures

#### Quantitative Survey

Participants completed a series of questions developed by the research team for this study, adapted from the Pew Research Center’s Internet and American Life Project survey [10]. To assess overall smartphone/device use, participants were asked to rate the frequency with which they engage in 14 behaviors using their smartphone or mobile device (eg, send or receive email, access a social networking site, look up health or medical information). Response options were on 5-point Likert scale from “never” to “every day”. They were also asked to rate how difficult it would be to give up their smartphone or mobile device on a 4-point scale ranging from “not at all hard” to “very hard”. For more detail regarding mHealth apps, participants were asked to indicate all categories of health-related apps (eg, exercise/fitness, diet/calories, medication management) they currently have on their smartphone/device and the names of the specific apps, if any. Participants also provided demographic data (eg, age, income).

#### Qualitative Interview

A trained research assistant administered a semi-structured interview, using a naturalistic inquiry approach with open-ended probes on the following topics: experiences with general and health-related apps, likes and dislikes of previously used apps, current use of apps in relation to CF management, and perspectives on mobile privacy and security. Participants were also prompted to discuss their thoughts about the following potential features of an app for CF management: medication/treatment reminders, tracking completed medications/treatments, earning rewards or prizes for completing medications/treatments, communicating or exchanging messages through the app (with the CF treatment team and family members), social networking with other people with CF, behavioral comparisons or competitions with other people with CF, games related to CF management, and any other app features they would like to see developed.

### Data Analysis

Descriptive data were calculated from the quantitative Web survey using Stata software, version 8.2, and qualitative data were coded from interview transcripts using NVivo software, version 10. To identify themes and develop an initial coding guide, five interview transcripts were collaboratively reviewed by the research team, which consisted of three clinical health psychologists, one clinical psychology graduate student, and one research coordinator. Two study team members coded the remaining transcripts using the initial coding guide. Discrepancies and coding scheme modifications were resolved through group discussion in an iterative fashion, repeated every five interviews until thematic saturation was reached. Inter-rater reliability between coders was determined by percent agreement and **kappa** coefficient.

## Results

### Participant Characteristics

Of the 16 study participants, 15 completed the Web survey, the characteristics of whom are summarized in Table 1. Participants reported that they used their smartphones/mobile devices to do the following activities every day: 93% (14/15) send or receive text messages, 80% send or receive emails, 80% (12/15) access the Internet, and 73% (11/15) access a social networking site. In addition, 64% (9/14; one missing response) and 47% (7/15) reported frequent use of gaming apps and real-time chat apps, respectively. Approximately one-half of participants (53%, 8/15) reported currently having at least one health-related app on their smartphone/device, the majority of which were related to diet or exercise.

**Table 1:** Participant characteristics (n=15)^a^.

Demographic characteristics	n (%)
Age, years, mean (SD) (range)	30.2 (5.9) (21-43)
Race, % Caucasian	15 (100%)
Gender, % female	7 (47%)
Marital status, % married/partnered	11 (73%)
Education, % college degree or beyond	11 (77%)
School, % currently attending	2 (13%)
Work, % full or part time	11 (73%)
Annual income, % ≥US $100,000	9 (60%)
Insurance status, % private	12 (80%)

^a^One of the 16 study participants did not complete the online survey.

### Qualitative Themes

A total of 16 interviews were conducted before thematic saturation was reached. Of the 16 interviews conducted, 15 transcripts were available. For one interview, the recorder failed but detailed notes were reviewed for content. Agreement between the two primary coders was high (97.3% agreement, SD 3.8) and inter-rater reliability was acceptable, in the moderate range (Cohen’s κ=47%, SD 0.43).

Participants discussed their preferences and concerns for potential future mHealth apps to promote adherence to CF treatments. One of the clearest overarching messages was the desire for an app tailored to the unique, complicated experience of having and managing CF. For example, one 24 year-old female said, “…the chronic illness community as a whole is kind of lacking apps geared toward them because we live a completely different lifestyle than someone who is not ...chronically ill… who doesn’t have the [same] kind of daily medical struggle or regimen”. A common challenge of using available health-related apps is that they may or may not fit with CF treatment goals. For instance, nutrition or diet apps are typically designed for weight loss, which is in stark contrast to CF nutrition guidelines that call for high calorie, fat, and sodium intake and often weight gain. The same respondent explained, “I found that most of those are geared towards losing weight and I wanted to be gaining weight and…it wouldn’t…let me input a higher goal weight for me…I find that a lot of…health-related apps are generally limited to the general public”.

Within the context of a “wish list” for features designed for CF-specific mHealth apps, four cross-cutting themes representing the key functions that study participants would find useful or appealing in a CF-related app emerged from the coded transcripts: (1) information at one’s fingertips, (2) automation of functions and integration with other technologies, (3) communication with health care providers, and (4) socialization within the CF community (described below, with representative quotes in Tables 2-5). Of note, there was not unilateral agreement about the potential benefits of each theme, and we present quotes reflecting both preferences and participant concerns about each theme. Participants also discussed strategies to enhance motivation through an app and shared their opinions about app design issues (eg, navigation, security, customization). Finally, participants shared ideas for specific functions or features of a CF mHealth app that they would find useful in their everyday CF management (Table 6).

#### Theme 1: Information at One’s Fingertips

One common preference of study participants was to have a central, accessible resource for information about CF in an mHealth app (Table 2). Participants were interested in accessing information on three levels: educational, personal, and outcomes. Because most educational or informational health resources currently available through apps are designed for the general population, providing information specific to CF was highly valued (Quote 2A). Participants also emphasized wanting easy access to their personal medical data, such as recent lung function values, current prescriptions, and hospitalization history, either synced from an electronic medical record or manually inputted for later reference (Quotes 2B-C). However, some people raised concerns about privacy issues when storing all health data in a central location (Quote 2D). Many were also interested in an app tracking their CF management behavior and health status over time to identify trends and associations between adherence and personally relevant outcomes (eg, physical symptoms, lung function, mood). Potential benefits of behavior tracking included giving insights into their own self-management patterns, increasing motivation, and facilitating informed communication with health care providers (Quotes 2E-F).

#### Theme 2: Automation and Integration

Many participants emphasized the importance of obtaining maximum useful output from an app with a minimal amount of input required (Table 3). They wanted “smart” functions that would passively capture data, thus requiring less input, and be highly relevant and responsive to their circumstances. For example, respondents recommended medication alarms that would adjust to changes in the regimen or their current activity or daily schedule (Quotes 3A-C). Many were also in favor of apps to automate the process of ordering prescriptions or refills for specialty medications, which often requires substantial time, paperwork, and coordination between providers and pharmacies (Quotes 3D-E).

**Table 2:** Participant preferences, concerns, and illustrative quotes regarding *Information at One’s Fingertips* theme.

Preference		Concern	Quote
**General CF information**			
	Educational resource for self and others (family, friends)	Generic information not useful, must be CF specific	A: “Sometimes I’ll [wonder when] something happens health-related to me, ‘Is that normal for everyone or…is that happening to me because I have CF?’ And it’s hard to find particular sources where I can find that out.” [Age 35, Female]
**Personal CF information**			
	Central, accessible storage for personal CF data (eg, medical history, prescriptions)	Privacy, data security	B: “I think that CF can be kind of overwhelming and it’s really nice to have one central location to keep important information and data.” [Age 34, Female]
			C: “Whenever you go to [a] doctor [they ask], ‘What’s your current list of medications?’…It’d be nice to have the whole history…and then have a place for notes for how well it worked.” [Age 48, Female]
			D: “I am not going to put medical information in one place unless I know that I can control who sees it.” [Age 31, Male]
**Individual behavioral and health outcomes**			
	Self-monitor adherence and health status data over time	Cannot guarantee accuracy of self-report, risk for dishonesty	E: “If you had an app that [tracked medication] doses…that said I missed 16 doses [on] 16 mornings…that might be a little bit of a wake-up call for me.” [Age 38, Female]
			F: “I like being able to see…where I fall on the [PFT] chart and correlate that with how I’m feeling…so if this app can track when I do my meds, when I do my treatments, and I correlate it to how I’m feeling maybe I’ll discover something like, ‘Oh, I feel the best when I do my vest at 1 pm instead of 8 pm.’ ” [Age 24, Female]

**Table 3:** Participant preferences, concerns, and illustrative quotes regarding *Automation and Integration* theme.

Preference		Concern	Quote
**Alarms and reminders for treatments**			
	Reminders for new, unusual, cyclical prescriptions	Don’t need alarms for common or routine treatments	A: “Last week they had me on a medication that I am not normally on, and I had to take that twice a day, so something [to remind me] might be helpful because…[for the medications I am normally on] I pretty much know the drill I’ve been taking them my whole life, but for stuff like that where it’s not part of my normal routine that could be helpful.” [Age 23, Male]
	Motivating or persistent alarms	Alarms can feel like nagging, are easy to “snooze” or ignore	B: “It needs to find the right line between annoying and not annoying…it needs to be just the perfect amount of annoying that I’ll actually do it.” [Age 24, Female]
	Well-timed reminders	Alarms that sound at inconvenient times	C: “The problem with alarm apps is that they tell you once and then you can…snooze…or just end it, and…if there’s a problem with taking the medication right then, that’s not good. For instance, if you’re driving you snooze and 5 minutes later it goes off again and you’re still driving…it would be really good if it could…[remind you later when] you’re not moving anymore.” [Age 30, Male]
**Pharmacy refills**			
	Automatic, triggered by available medication data (eg, new or previous fills, adherence data)	Refill barriers: forgetting, late requests, time-consuming calls, pre-authorizations	D: “The biggest thing for me would be the ability to manage medications…all in one place …If I need to refill prescriptions, … being able to potentially do it straight from my phone … with the ability to tie into … wherever I get my medicine from so that when I do need to renew it or refill it, it's … the click of a button or scanning of a bar code in the phone…If I could have one central location that had all of my prescriptions that dealt with cystic fibrosis in one spot and I could refill them from that spot, that would make life a lot easier.” [Age 31, Male]
			E: “A lot of people need reminders when they need to refill their prescription so…keeping track of how many [doses of medication] do I have left?…Keep track of when I filled that and automatically populate 30 ampules…then it counts down every time you use, being able to track when you’re supposed to order your medication again.” [Age 27, Male]

#### Theme 3: Communication

Opportunities to seek or share medical information with other people were frequently mentioned (Table 4). Participants were interested in using an mHealth app to communicate with their health care provider between clinic visits and to improve care by giving providers access to personal CF data through the app (Quotes 4A-B), although concerns were raised with the quality of app-based communication compared to live or telephone contact (Quote 4C). On the other hand, many perceived health or behavior tracking as a mechanism to enhance communication with their CF providers by providing relevant data (Quotes 4D-F). Participants also discussed ways that an mHealth app could facilitate efficient conversation within the health care system, such as to promote care coordination among different providers or between prescribers and pharmacies (Quotes 4G-H). Finally, some respondents envisioned mHealth apps that could promote communication within families, for example to help parents monitor adolescents’ CF treatment adherence, although this was less appealing for adults (Quotes 4I-K).

#### Theme 4: Socialization

Study participants often saw a social networking component as a possible solution to the widespread sense of isolation among people with CF (Quote 5A; Table 5). Respondents almost unanimously agreed that a closed social network separate from existing public social networks or platforms was important, not only for privacy, but also to build a community of people with similar experiences (Quotes 5D-F). Many emphasized the uniqueness of their individual experiences or CF genotype and wanted highly filtered networks based on their demographic and health status to maximize the benefit of socializing, which may not otherwise be possible given their rarity of CF (Quote 5G). Social networking was perceived to be appealing for family members or partners of people with CF, as well (Quote 5H-I). While several respondents saw potential benefits in sharing personal experiences and data via a social network (Quotes 5J, L), concerns were raised about privacy (Quotes 5M). Many respondents also discussed the risk of feeling saddened by the high rate of mortality and illness in the CF community and wanting to limit contact with others to avoid these reminders (Quotes 5B, C, K).

**Table 4:** Participant preferences, concerns, and illustrative quotes regarding *Communication* theme.

Preference		Concern	Quote
**With medical team**			
	Contact with CF providers between visits	May not be as responsive as traditional telephone or email contact, don’t want to replace human contact with providers	A: “I’m not always in a place where I can call them, so if I can just shoot a text…that would be convenient…If they want me to do something out of the ordinary...I want to [ask], ‘How exactly did you want me to do this?’ ” [Age 23, Male]
			B: “If there is an impending exacerbation coming up, being able to communicate with them about that and do a…plan of action.” [Age 35, Female]
			C: “In the everyday world [electronic communication] just seems to be replacing talk and conversation and you know, communicating that way, I don't want that to happen [with my doctors].” [Age 35, Female]
	Sharing health data with CF providers can facilitate informed conversations during medical visits	Discomfort with medical team having high level of monitoring ability	D: “Obviously they’re the experts, but…it’s empowering to…go into an appointment with salient data.” [Age 34, Female]
			E: “If you started to get sick and the nurse says, ‘Well how have you been doing…taking these medications?’…Being able to say, ‘Yeah, here is what I’ve been doing.’ …would be a nice function to have.” [Age 31, Male]
			F: “I don’t know if I’d want it but…it would be good for them to see…how much you did a treatment in a week. I feel like they probably should see that but you might not want them to.” [Age 21, Female]
**Within health care system**			
	Care coordination between CF team and subspecialists or other providers		G: “My regular doctor…puts me on [a medication] for high blood pressure…then I need to go talk to my CF doctor to make sure this isn’t going to screw up my [CF medication] so that they know what I’m on…if there’s a way…when I’m up at CF clinic and they change my regimen you could use the app and it will automatically…send it to my regular doctor…so I am not manually having to remember to send stuff back and forth.” [Age 48, Female]
	Between pharmacy and CF team to submit prescriptions, authorize refills		H: “I could have a list of medications and submit a refill request to my doctors [through the app]…ideally it would integrate with the pharmacy app…and have the ability to say, ‘You’re out of refills on this,’…and it could notify my doctor [directly] instead of notifying [my pharmacy] to notify my doctor.” [Age 28, Male]
**With family**			
	Parents would find it valuable to help monitor teens’ adherence	While potentially helpful for others, adults did not need a new communication channel	I: “It might be good for parents to…be able to see when the medication is being taken and when the treatments are being done.” [Age 30, Male]
			J: “I would use other more direct messages.” [Age 28, Male]
			K: “I would probably continue to communicate with them the way I already do.” [Age 33, Female]

**Table 5:** Participant preferences, concerns, and illustrative quotes regarding *Socialization* theme.

Preference		Concern	Quote
**With other people with CF**			
	Novel opportunity to network, given prohibitions on face-to-face contact	Could feel discouraged or guilty seeing others doing better or worse than you	A: “Because people with CF can’t be in the same room as each other,…being able to see someone else with CF is much more profound than just exchanging emails with some anonymous person.” [Age 28, Male]
			B: “I don’t like hearing about CF people that aren’t doing well. I have a hard time distancing myself from it. It’s hard having to filter through all this sadness to get kind of connected with someone.” [Age 26, Female]
			C: “I didn't really like it only because some people had it worse than me and if it kind of brought me down because I felt like this is where I'm heading and I just didn't like that…So I don't know if I'd really want to talk to any other people with CF, I don't want to like be depressed.” [Age 32, Male]
	Access to people with similar experiences	Variability within CF does not guarantee shared experiences	D: “At some point in our lives, we feel inadequate and we're not as good as normal people so this gives us our own sense of community that way we can relate to…people who are like us.” [Age 27, Male]
			E: “It's always nice to know another mom that has CF, like, ‘How do you juggle getting your child out to school versus doing your treatment?’, you know that sort of same situation.” [Age 38, Female]
			F: “The ability to ask questions to a larger community regarding…issues or problems that you may be having from a CF standpoint…A CF to CF community...[is] what I found lacking, that I didn't have when I was growing up.” [Age 31, Male]
			G: “There’s like 1800 different kinds of CF right now and they’re still discovering more, so it’s never going to be a specific thing unless you could somehow log…your exact genes…and then maybe connect with people that have the exact same ones.” [Age 30, Male]
	Social support opportunities for families, partners, caregivers of people with CF	Challenge to maintain other person’s privacy or anonymity if organized through app of person with CF	H: “I think that support for the family and friends is important…for people who have CF…talking to significant others of people who have CF.” [Age 28, Male]
			I: “Something to…connect friends and family so that they understand the disease a little bit more, and maybe they can…connect with you on a different level.” [Age 32, Male]
**Comparisons for motivation**			
	Self-monitoring in context of others with CF	Discouraging if not meeting goals, doing worse than others	J: “To build a community…would help provide people a better incentive…more of the ‘We’re in this together.’ Someone else has worked 12 hours a day but they’re still doing their medicine, you know I can.” [Age 28, Male]
			K: “Sometimes you don’t want to be comparing yourself to other CF-ers because it is depressing… like, ‘She has worse lung function than me but she is never sick, why is that?’…It can be tough because no two cases are the same and yet we all…have similar therapies so it can be frustrating to see a better outcome than yours if you are doing all the same things if not more.” [Age 24, Female]
	Potential for motivation and reinforcement from peers	Concerns about anonymity and privacy, ability to be identified	L: “If you’re going to see somebody that is doing pretty well and you can…talk to them about how they handle it or what their regimen is, then maybe you can pick up things from them or share with them what you do.” [Age 35, Female]
			M: “If I were to have my data compared to the whole…I wouldn't want them to say, ‘Oh there's someone named [removed] that didn't do treatments today.’ ” [Age 26, Female]

### Other Potential Motivators

In addition to the four themes discussed above, participants were also prompted to discuss other possible app features that could be potential motivators. Opinions were mixed regarding the usefulness of gamification, behavioral comparisons, and competitions to enhance motivation among app users. One point of agreement was the importance of comparing adherence behaviors rather than clinical indicators (eg, lung function, weight), because individuals have more control over their behaviors than their health outcomes. Some participants discussed potential benefits of comparing their adherence behaviors with others, such as, “it may be helpful…if other people are able to share how they’re able to do it or…motivate you to do a better job” [age 38, female]. However, many did not imagine feeling motivated by comparing their data or competing with other people, from whom they may be very different. One respondent described her disinterest in competitions, “I’m shooting for the best I can be, not the best that other people can be” [age 33, female]. Others raised concerns about negative feelings, such as guilt, from comparing oneself to others: “If I see someone who [has a very poor disease state], I’m going to start feeling guilty because I do not have that” [age 27, male]. Another participant discussed possible discouragement, “you wouldn’t want to make somebody feel horrible about themselves if they’re maybe not…doing everything they should be doing” [age 35, female].

The use of prizes to motivate health behavior was also controversial. Some respondents were eager to earn rewards for completing CF treatments, especially those that were tailored to their interests or that benefitted the CF Foundation, while others did not feel that material prizes would sufficiently motivate them. One 24 year old female participant expressed her ambivalence about rewards, “Day after day it’s hard…it’s your health and your life, and that should be motivation enough, and well…sometimes, gosh darn it, it isn’t.”

### App Design Recommendations

Participants shared suggestions for app design features that would meet their needs and preferences for a CF management app (Table 6). For example, to address preferences related to automation and integration, study participants described app features that would automatically suggest optimal times for CF tasks based on all available information by syncing with other apps or using other smartphone technologies such as motion detection or other sensors. Some participants imagined systems to automatically provide medication reminders or refill prescriptions using data stored in the calendar or pharmacy app. Another described the potential benefits of video chatting for communication with others with CF, “The video thing that is a way for CF patients to interact with each other kind of face-to-face, which…we’re not allowed to do” [age 27, male].

Participants raised several points about the app design and usability. A central issue for many was ease of use, with minimal burden or effort to input medical data or set up scheduled alarms. Participants described frustration with other apps having “tedious” data entry and access processes [age 33, female] that limited the app’s usefulness. One participant acknowledged the long-term benefits of entering personal data up-front, “that may take …back end [effort] on the user’s part, like on my part it may take me importing account information… but I don’t mind doing that if it makes it easier in the long run” [age 31, male]. Yet the overarching sentiment was to use automated processes for passive data collection to reduce requirements for user effort.

Clear, intuitive navigation was important, with the ability to easily search data within the app. Participants wanted graphical displays to be presented in digestible, easily understood, and mobile-friendly visual formats. Simple features, such as a basic alarm or medication reminder system, were generally not of interest; rather, participants favored app designs that integrated multiple functions within a single, cohesive program. As one participant described her vision, “I don’t want a bazillion apps…it would be so great to have an all-in-one where, I could keep track of my calories and also my exercise, and also, ‘Well, I did saline three times today, gold star for me.’…all compiled into one [app] so I can quickly look at this one app and see an overview of my progress or my lack of progress…so if it could be presented in an orderly and efficient fashion all in one place would be great” [age 24, female]. However, with so many possible features within a single app, several participants also emphasized the need for customization options, such as selecting which features to use and privacy settings. For example, some participants were comfortable with their CF providers being able to track their adherence or refill data, while others wanted to limit access. Some were interested in social networking, while others would prefer not to use that feature. Given the heterogeneity in participant preferences and concerns about app features, the option to turn individual features on and off will be very important to ensure acceptability and use of an app. Finally, a common recommendation was to sync the app across multiple devices so that the data and functionality could be updated and accessed not only from a smartphone, but also from other mobile and desktop devices via the Internet and the cloud.

**Table 6:** Suggested app features related to cross-cutting themes.

Feature	Theme			
	Information at One’s Fingertips	Automation and Integration	Communication	Socialization
Searchable CF-specific medical wiki-resource with video clips and interactive resources	●			
Sensors to assess lung function (spirometry), sweat chloride, medication, or treatment adherence	●	●		
Graphical displays of adherence behaviors and outcomes (eg, symptoms, spirometry, mood) over time, visualize links between treatments and outcomes	●		●	
“Smart” goal-setting guidance based on medical team’s treatment plan and tracked adherence or outcome data		●	●	
“Genius” suggestions for personalized behavioral incentives (eg, for adherence) based on Web-browsing or purchasing history		●		
Timed serial alarms for consecutive series of treatments		●		
“Genius” treatment scheduler / adherence prompt based data from other apps: personal schedule (calendar events), current location (GPS), activity level (motion detection)		●		
Recognize new or cyclical (month-on, month-off) prescriptions (eg, via linked pharmacy app) and automatically set up reminder/alarm		●		
Automatic medication refill requests sent to pharmacy based on passively collected adherence data (eg, sensors on medication packaging, self-reported administrations)		●	●	
Schedule and sync medical appointments with existing calendars (eg, Outlook or Google calendar appointment requests)		●	●	
Barcode/Q-code scanner to access CF-specific information about medications or food, to track adherence to treatments, to request prescription refills	●	●	●	
Direct contact buttons (call, email, text) to reach CF team, one for emergencies, one for routine/non-urgent contact			●	
Video chat – with CF team, pharmacy, other people with CF		●	●	●
Two-way messaging with CF team – Q&A with providers, providers contact patient via app if behavioral/outcome monitoring indicates health concern		●	●	
Connect with others with CF with similar experiences or characteristics via searchable database of participant profiles				●
CF-specific social networking platform, can opt into sharing personal data to public sites		●		●

## Discussion

### Principal Findings

Based on the thoughtful and realistic input of potential end users (ie, smartphone-using individuals living with CF), this study provides direction for future development of patient-oriented mHealth apps for CF adherence promotion. It should be noted that participants were asked to comment on their experiences and their “wish lists” and not an already developed app. This afforded them the opportunity to be blunt about preferences without concern about negatively commenting on the work of the investigators. Moreover, in contrast to other studies on patient perspectives [39,40], the primary topic of inquiry was desired design features, not educational content. Study participants largely expressed excitement and interest in potential future mHealth apps designed to facilitate CF management. Participants emphasized the importance of having easy access to health information, automating app functions to avoid creating additional burden, integrating smartphones’ technological capabilities to increase efficiency for time-consuming disease management tasks, and facilitating timely and meaningful communication with others with CF or health care providers. However, important concerns about the usefulness, appeal, and security these issues were also raised. Given the opportunity to brainstorm future app features, respondents described inventive, creative, yet realistic functions that have the potential to ease the burden of daily disease management and facilitate healthy behavior patterns. While this study focused on individuals with CF, the key themes, concerns, and ideas raised are likely relevant to people with a number of chronic medical conditions with complex and burdensome management regimens.

### Common Themes in mHealth App Preferences

Several of the themes voiced by this study’s participants echoed the preferences of other research related to user preferences for mHealth apps. Perhaps most universal are the desire for low-burden, high-payoff apps and the need to be able to customize app content and features to fit individual health needs and wants [27-29]. Across people with and without chronic conditions, these were nearly universal and should be applicable to most mHealth app designs.

Specific content and functionality also had some similarities to findings from other research. Participant interest in communicating via an app with health care providers in this study was consistent with similar themes in studies with people with type 2 diabetes [27] and HIV [29], highlighting the importance of patients desiring greater access to their health care team. The other studies did not discuss using the app to facilitate communication and cooperation among health care providers and other services such as pharmacies. However, this was suggested by the participants with CF, perhaps due to the multiple systems involved in the treatment of CF and the burdensome process of ordering specialized medications and equipment. Qualitative research to guide mHealth apps for more general health behaviors has called for automated processes to track and provide feedback about physical activity and diet [28,30]. This is similar to the adherence behavior monitoring components discussed in this study. The ability of smartphones to passively collect, store, and synthesize behavioral data may be helpful across numerous health behaviors and populations.

Like the current study, the study on apps for individuals with HIV reported a theme related to automated medication prompts based on current contextual sensing (eg, geolocation) [29], while individuals without chronic conditions were less optimistic about how this technology could support their health behavior goals [28]. It may be that the highly structured, time-intensive, and location-specific (eg, specialized equipment at home or medical facility) daily demands of chronic condition treatment regimens benefit more from personalized prompts than do the more flexible health behaviors of generally healthy adults. In contrast to the samples of people with CF or type 2 diabetes [27], young adults without a chronic medical condition were less interested in social networking opportunities around supporting health behaviors such as eating or exercise [28]. Social networking may be particularly attractive to people with chronic medical conditions who feel alone in their disease or who are seeking guidance or support from others in similar situations. Individuals without chronic health conditions may receive adequate support from other sources and not desire health behavior-specific support from other online users. Alternatively, people with chronic conditions may not feel comfortable sharing their health behavior data via general social networks that reach people to whom they may not have disclosed their diagnosis; sharing information about health behaviors may be much more socially acceptable for people without chronic conditions (eg, tweeting today’s bike ride distance, posting a photograph of tonight’s healthy dinner) [28]. It appears that particular features like context sensing and social networking have greater appeal for chronic condition management and should therefore be considered independently for each population developing its own mHealth app.

### Making Use of Available Technologies

Ever-evolving smartphone technologies make many of the recommendations and themes of this study’s participants plausible. Given the prevalence of social networking in general and the CF-specific challenges of social isolation, it is unsurprising that app-based socialization with other people with CF was a major focus for many participants. The potential for feeling isolated is elevated in CF due to the guidelines to reduce risk for infection by eliminating close contact with others with CF [34]. Widespread social networking and communication features common to smartphones, including text, talk, and video messaging, can be easily employed to create previously impossible opportunities for socialization and support among people with CF and their families. Similarly, many participants imagined the benefits of having an app that could reduce the arduous and time-consuming tasks of refilling complex prescriptions or that could enhance care coordination between multiple medical providers, pharmacies, and insurers. By capitalizing on existing technologies, these challenges could be easily resolved. For example, a personal mHealth app could sync with apps for pharmacies and integrate with patient-provider electronic medical record portals to automate these processes and efficiently use already available resources and infrastructure.

Internal and external sensors in smartphones are also increasingly available for use with future mHealth apps. For example, mHealth systems have been developed that use machine learning, or the context sensing capabilities of smartphones, to provide psychological interventions in timely, relevant, and highly personalized ways [22,41]. Sensors that are native to smartphones, including GPS geolocation, motion detection, voice recognition, and calendar event monitoring, are available for coordination with mHealth apps. External sensors could also be integrated to detect other data about individuals’ behavior or context, such as physical activity via electronic pedometers or medication adherence via electronic medication monitors [42]. For people with pulmonary conditions like CF, data from an external peak flow meter could be used to track and provide feedback on patterns of lung function [43]. Although these technologies exist, they have not been maximally used for personalized behavioral interventions related to chronic illness management [21-24]. Thus, there remains great potential to take advantage of sophisticated tools in the development of patient-centered mHealth apps. Moreover, even as smartphones have growing capability to collect numerous types of monitoring data, little attention is being given to the most effective and user-friendly ways to provide feedback to both the user and health care team. As noted by many participants in this study, tracking health behaviors with clear graphs and visualizations is likely of great interest and benefit.

### Stakeholder Input in the Design Process

To successfully create and evaluate the impact of mHealth apps for chronic illness management, many stakeholders need to be included from the start and throughout the development and evaluation process [26]. The themes of integration and ease of use identified in this study highlight the need for apps to provide a clear perceived benefit to the user in order to keep them engaged. Although many mHealth apps are available, repeated use is extremely low [44-46]. For example, an analysis of a dietary self-monitoring app with photography and feedback found 2.6% of users were considered “active” and user feedback was rare, with only 15.4% of uploaded photos receiving a single “like” from other users [44]. It is critical to create apps that are not only initially appealing but also maintain engagement over time, in order to have optimal benefit for users or improve health outcomes. As evidenced in the current study, end users (in this case, individuals with CF) are critical to informing the direction, content, format, and focus of eventual apps [25,26]. Including the target population end users in all stages of mHealth development allows developers to harness patients’ experiences, perspectives, preferences, and—perhaps most underutilized and undervalued—their creative imaginative solutions to the everyday challenges with which they are intimately familiar. When recruiting potential end users into a study like this one, it is critical to include participants with varied characteristics in order to maximize the likelihood that their feedback will apply to many types of people.

A common theme in this study was the desire for integration of app functions across various health care providers and services. To achieve an integrated product, it is recommended that app developers should coordinate with representatives from multiple sectors of the health care system (eg, pharmaceutical companies, pharmacies, insurers, medical providers). To ensure that the app features are integrated in a way that is consistent with behavioral theory and evidence [47], psychologists and behavioral scientists should be consulted [20]; additionally, they could advise on research design and evaluation. Finally, technology experts, including computer scientists and engineers, and experts in privacy and security, are also crucial to executing the app or mHealth program effectively. Not included in this study, but also important to survey, would be family members and other supporters of individuals with the chronic condition for whom the app is designed, as well as health care teams, pharmacists, and insurers whose activities could be impacted or touched by app features such as those described in this study.

### Strengths and Limitations

As with all mixed-methods research, this study has strengths and limitations. This study gathered rich qualitative data and was able to quantitatively characterize the demographic, clinical, and behavioral profiles of a sample of adults with CF, a chronic condition with a complex and burdensome treatment regimen. The qualitative data were coded by two independent raters, and the inter-rater reliability coefficient kappa was in the moderate range. The kappa statistic takes into account the possibility of agreement due to chance. While this is useful for categorical data, it relies on the assumption that a coder who is unsure what code to select will rely on chance or guess randomly. This is highly unlikely for qualitative coding because coders often have some background information to guide selection and thus results in a conservative underestimate of agreement for qualitative data [48]. Thus, paired with the high percent agreement between raters, the moderate kappa is acceptable.

Participants were varied in age, disease status, and adherence behaviors, yet all were smartphone users who would be potential consumers of an mHealth app. Though small, the sample size was adequate for the purpose of this study to inform directions for mHealth app development with a user-centered design, and we were able to identify a number of common themes and to reach saturation. Participants were actively recruited, which resulted in a sample that better represented the clinic population than had we relied on participants to volunteer in response to advertising. Nevertheless, individuals who agree to participate in research may be more motivated or knowledgeable about their condition than those who decline or do not respond to invitations to participate, which may have an impact on study outcomes. While CF is an exemplar for a complicated management regimen, it is a rare disease with many unique aspects that may not generalize to other illnesses.

### Conclusion

Finally, apps create a large amount of data for both patients and providers, but little is known about how the data will be received and used by providers in clinical care. It will be important to include health care providers and other stakeholders in future research to determine their needs, preferences, and uses of mHealth apps for chronic condition management.

## Abbreviations

CF: cystic fibrosis
